# Role of Biomaterials Used for Periodontal Tissue Regeneration—A Concise Evidence-Based Review

**DOI:** 10.3390/polym14153038

**Published:** 2022-07-27

**Authors:** Jothi Varghese, Anjale Rajagopal, Shashikiran Shanmugasundaram

**Affiliations:** Department of Periodontology, Manipal College of Dental Sciences, Manipal Academy of Higher Education, Manipal 576104, India; anjalermenon@yahoo.com (A.R.); shashi.ks@manipal.edu (S.S.)

**Keywords:** periodontal regeneration, bone grafts, calcium phosphate grafts, autologous platelet concentrates, nanohydroxyapatite bone graft substitutes, guided tissue regeneration substitutes, scaffold designs

## Abstract

Periodontal infections are noncommunicable chronic inflammatory diseases of multifactorial origin that can induce destruction of both soft and hard tissues of the periodontium. The standard remedial modalities for periodontal regeneration include nonsurgical followed by surgical therapy with the adjunctive use of various biomaterials to achieve restoration of the lost tissues. Lately, there has been substantial development in the field of biomaterial, which includes the sole or combined use of osseous grafts, barrier membranes, growth factors and autogenic substitutes to achieve tissue and bone regeneration. Of these, bone replacement grafts have been widely explored for their osteogenic potential with varied outcomes. Osseous grafts are derived from either human, bovine or synthetic sources. Though the biologic response from autogenic biomaterials may be better, the use of bone replacement synthetic substitutes could be practical for clinical practice. This comprehensive review focuses initially on bone graft replacement substitutes, namely ceramic-based (calcium phosphate derivatives, bioactive glass) and autologous platelet concentrates, which assist in alveolar bone regeneration. Further literature compilations emphasize the innovations of biomaterials used as bone substitutes, barrier membranes and complex scaffold fabrication techniques that can mimic the histologically vital tissues required for the regeneration of periodontal apparatus.

## 1. Introduction

The holy grail of periodontal therapy is the regeneration of the attachment apparatus lost due to periodontitis. This noncommunicable chronic inflammatory disease of multifactorial origin is known to cause damage to soft tissue and induce destruction of the periodontium [1]. The loss of alveolar bone remains the hallmark of periodontal disease progression, which is a therapeutic challenge for clinicians across the world. This pathogenic mechanism is facilitated by the biologically active substances within the subgingival plaque that induce a local inflammatory response in the supporting structures of the periodontium [2]. In addition, the host immune cells propagate various proinflammatory cytokines, i.e., PGE2, IL1 and RANKL, which stimulate the resorptive activity of the osteoclast favoring bone loss [3]. Furthermore, the neutrophils and macrophages indulge in the secretion of matrix metalloproteinases (MMPs), which degrade collagen and other components of the extracellular matrix, leading to destruction of connective tissue. These immunopathologic events manifest as periodontitis, which does not necessarily result in tooth loss. The severity of tissue damage and the rate of progression of disease are governed by various factors such as genetics, environmental and systemic, rendering it a multifactorial, chronic inflammatory disease.

The successful outcome of periodontal regeneration in combination with various therapeutic modalities (scaling, root planing, curettage, flap surgery) are evident in the literature [4]. However, these approaches also present limitations without the use of adjunctive materials that could provoke tissue regeneration in periodontally compromised areas. The recent years have visualized and explored considerable progress in the field of biomaterials for tissue engineering, which includes the use of barrier membranes, osseous grafts, growth factors and the combination of these procedures [5].

Bone grafts can be broadly classified as autografts, allografts, xenografts and alloplasts (synthetic). Among them, bone replacement synthetic grafting procedures are more pragmatic even though autologous bone grafts provide a better biological response. There are a few disadvantages, such as additional surgical time and donor site morbidity, that are overthrown by artificial bone substitutes. Osseous grafts are derived from either human, bovine or synthetic sources. The human or animal sources may have issues related to ethics or disease transmission. Hence, there is a demand for artificial/synthetic bone substitutes, with biological stability, biocompatibility and negative immune response having similar or better efficiency, which could be procured less expensive than allogeneic and xenogeneic substitutes [6,7].

The synthetically developed bone substitutes used in clinical practice are broadly classified as polymers, metals and ceramics. However, these bone substitutes exhibit few drawbacks related to poor mechanical properties, low biocompatibility and poor adhesion to human tissues [8]. In order to overcome these limitations, calcium-phosphate-based ceramics were developed due to its remarkable properties, the most crucial being its similar composition to bone minerals and its origin being in abundance in human bones. These materials stimulate cellular activities, resulting in the formation of a unique CaP interface. Additionally, CaP materials have a three-dimensional design, which permits their adherence to endogenous bone morphogenetic proteins within the human system. This property can induce osteoinductive properties [5]. Considering these distinctive features, calcium phosphate ceramics were widely used and probed extensively for their regenerative potential [9,10,11].

## 2. Calcium Phosphate Biomaterials

Hydroxyapatite (HA) is the most commonly used calcium phosphate bone graft material across the world. Its structure and composition are comparable to native bone [12]. Van Meekeren was the first to introduce synthetic bone graft with calcium sulfate in 1892. Since then, the term ‘bioceramics’ was documented and used as bone substitute agents in humans [13]. Hydroxyapatite bone substitutes are referred to as bioactive materials due to the osteoconductive characteristics that permit apposition and migration of osteoblasts [8,14]. The biocompatibility of synthetic hydroxyapatite embedded in the human periodontium was studied using TEM, and the authors found the osteoblasts on the HA surface triggered initial osteoid formation, which, in time, mineralized to complete dense bone. In 6 months, small apatite crystals were observed placed in the center, surrounded by larger crystals of synthetic HA [15]. Several studies have been conducted on hydroxyapatite bone grafts alone or combined with other bone substitutes and have gained significant clinical bone gain in all fields of dentistry for regeneration of supporting alveolar bone [16]. A recent systematic review based on applications of nanohydroxyapatite in dentistry concluded that it could be a favorable material for various dental treatments, while in implantology, it outperformed other materials used as coatings for titanium implants. Nano-HA improved bone regenerating properties compared to autologous bone grafts in tissue engineering. In combination with different scaffolds, notable results in periodontal treatment were seen [17].

### 2.1. Tricalcium Phosphate (TCP)

TCP is a resorbable form of bone substitute broadly classified into two phases, i.e., α and β, which are chemically similar but tend to perform differently based on their functional environment [5]. Considering its biocompatibility and osteoconductive features, β-TCP was used to treat intrabony defects and regenerative procedures, which revealed complete resorption of the osseous graft within a time interval of 6 months to one year [18]. β-TCP used alone or in combination with allografts, and xenografts have offered promising outcomes with reference to percentage gain in alveolar bone or with the development of mature bone in defect sites [19]. On the contrary, there were authors who achieved conflicting results in the percentage of bone fill to different regenerative procedures using β-TCP [20].

Biphasic calcium phosphate ceramics: These are a combination of two different CaP phases; a more stable phase (HA), which is more common, and a more soluble phase (β-TCP) in different proportions. Degradation of bone grafts is an important factor in bone formation. In order to increase biodegradation, biphasic calcium phosphate was developed for osseous defects. It is a bioceramic available in different formulations such as powders, granules and blocks for regenerating bony defects and is widely used across the world [21]. Wang et al. 2011 surgically created dehiscence defects in the alveolar bone of 12 beagle dogs. The defects were treated with BCP (40 HA/60 β-TCP) or with OFD. The results indicated that BCP augments periodontal regeneration in the above-mentioned defects [22]. Biphasic calcium phosphates have been shown to be bioresorbable with osteoconductive properties when the physiochemical characteristics are controlled [23]. The effect of BCP was explored on the differentiation and survival rate of osteoclasts in chronic periodontitis patients. The authors remarked that BCP limits osteoclastogenesis through promotion of the proteolytic cascade of apoptosis and displays favorable effects in combating alveolar bone resorption in chronic periodontitis [24].

### 2.2. Plaster of Paris/Calcium Sulfates

The literature records the use of calcium sulfates predominantly in the orthopedic specialty. However, in two different papers, Dreesmann and Peltier reported that calcium sulfates can be used effectively to fill and treat osseous defects [25,26]. Calcium sulfates possess beneficial characteristics such as bioabsorbability [27], osteoconductive properties, low immune reactivity [28] and enhancement of the migratory ability of fibroblasts [29]. The combination of β-TCP and calcium sulfates is commercially available as Fortoss^®^, which can be used solely without the use of a barrier membrane, with lesser surgical time and cost. All these features make it preferable for adjunctive use as regenerative material. Thus, clinical reports describe it as an alternative to nonresorbable membranes (e-PTFE), which are known to be time consuming [30]. A mixture of calcium sulfate and demineralized freeze-dried bone allograft (DFDBA) improved clinical results for class II mandibular furcation defects compared to the use of calcium sulfate as a single entity [31]. Micro- and nanoformulated crystalline forms of calcium sulfate were attempted for use as a possible treatment for intrabony defects. The authors concluded that nanocrystalline calcium sulfate presented significantly enhanced periodontal regeneration compared to the microcrystalline form [32].

### 2.3. Bioactive Glass

Several biomaterials have been introduced over the years. In the late 1960s, Hench and coworkers developed a group of surface-reactive glass ceramics, including bioactive glass, which gained popularity in periodontal and implant surgeries [33]. The key element of bioactive glass is its biocompatibility and capacity to act rapidly as a biomimetic mineralizer and match the natural human skeleton’s mineralizing traits [34]. This bioactive material consists of minerals that occur naturally in the body (silica (SiO_2_ (46.1 wt.%)), sodium oxide (Na_2_O (24.4 wt.%)), calcium oxide (CaO (26.9 wt.%)) and phosphorus pentoxide (P_2_O_5_ (2.6 wt.%))), and the molecular proportions of calcium and phosphorus oxides are similar to natural bone. A recent comprehensive review on bioactive glass applications deduced that the most common bioactive glass compositions (in particular, 45S5, S53P4 and borate-based glass 19-93B3) are versatile replacement materials, and their availability in different designs enhances their use in the treatment of human defects among clinicians [35].

Schepers and Ducheyne coined the term ‘osseostimulation’ to describe the process of bone formation in bioactive glass [36]. De novo bone formation using bioactive glass was studied using a periodontitis-induced model in monkeys. The authors affirmed that the new bone formed within the intrabony defects was situated at a site distant from the defect [37]. In another clinical study, the authors observed an active build-up of an osteoid matrix directly on the bone graft particles, which was suggestive of bone formation [38].

## 3. Autologous Materials as Regenerative Substitutes for Periodontal Regeneration

Researchers have developed many regenerative procedures over the decades to treat periodontitis [39]. However, the recent treatment modalities used either alone or in combination have limitations in producing true regeneration, especially in advanced periodontal defects [40]. This challenge has led to a trail to find the most suitable material that could regenerate the lost periodontium with greater chances of new attachment and fewer complexities.

**Figure 1:**
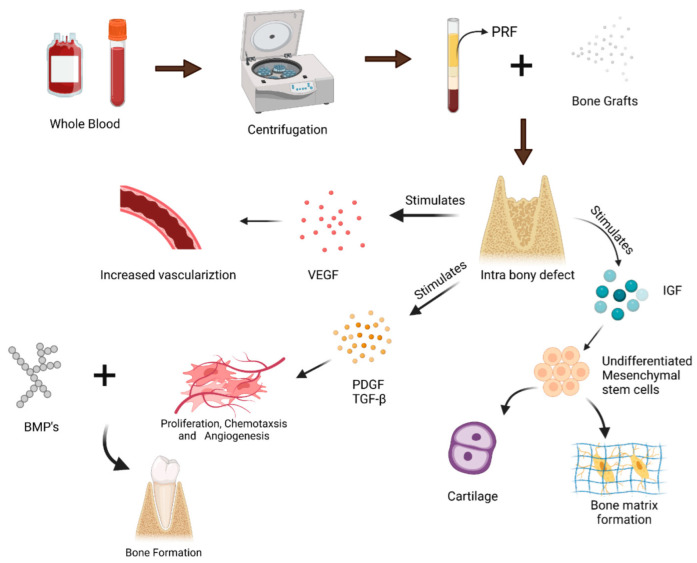
Sequential events that depict the bone regeneration after PRF application.

Clinical trials conducted in the field of regenerative medicine use allografts, xenografts, alloplasts and/or their combinations in view of the evidence related to their regenerative potential. Nevertheless, these mentioned bone graft resources have a major drawback of foreign body reaction or negative immune response, which impairs their use in periodontal defects [41]. Considering these limitations, clinicians prefer an autogenic biomaterial that could aid in tissue regeneration with minimal reaction when introduced into the human body. One such fibrin matrix, documented initially by Choukroun, had a blend of growth factors and cytokines, i.e., platelet-rich fibrin (PRF) [42]. This second-generation PRF clot prepared from a patient’s own blood after centrifugation develops into a three-dimensional strong fibrin scaffold that contains platelets, leukocytes and growth factors (platelet-derived growth factor (PDGF), vascular endothelial growth factor (VEGF), transforming growth factor-beta (TGF-β), insulin growth factor 1 and 2 (IGF-1/ IGF-2) and epidermal growth factor (EGF)), which are critical for tissue healing [43,44]. These biological proteins stimulate the periodontal ligament cells to differentiate into osteoblasts, which leads to bone regeneration [45]. This sequence of events is illustrated in Figure 1.

Initially, Serroni et al. conducted a clinical trial to study the additional benefit of leukocyte-platelet-rich fibrin (L-PRF) to autogenous bone graft compared to autogenous bone graft and open-flap debridement alone. The results of the study showed a better outcome with L-PRF for degree II furcation management [46]. In another randomized trial, Rexhepi et al. demonstrated that an inorganic bovine bone graft in combination with L-PRF was noninferior to a combination with a collagen membrane (CM) when managing unfavorable infrabony defects (IBDs) [47].

Different variants of PRF appeared in due course through variations in centrifugation protocols. This was originally prompted to investigate the intensified release of growth factors. One such development was advanced platelet-rich fibrin (A-PRF) introduced by Choukroun (2014), obtained by low centrifugal speed (1500 rpm, 14 min), which stirred the leukocytes to the bottom of the test tube for a more even distribution of neutrophils and unbound fibrin matrix. The neutrophils present in PRF have soft and hard tissue regenerative capabilities to direct monocytes to phagocytosis and produce proteases such as MMP9 for the wound healing process [48]. Additionally, the application of PRF for periodontal infrabony treatment exhibited a reduction in pocket probing depth and relative attachment levels [49]. Another study conducted by Lei et al. (2020) using a combination of A-PRF and concentrated growth factor (CGF) for periodontal defects displayed a continual and steady release of total growth factors over a 14-day period. The authors remarked that the combination of concentrates showed similar effectiveness in periodontal bone regeneration, with a potential benefit of improving GTR outcomes when used in intrabony defects [50].

Further development in PRF was the elimination of silica in glass tubes required for the formation of platelet aggregation and fibrin. Few health risks caused due to the presence of silica in these glass tubes were discussed [51]. Hence, attempts to develop PRF in titanium test tubes led to the advancement of platelet concentrates to T-PRF. The titanium present in the test tubes is passivated into an oxide layer within itself, which activates platelets and forms a thicker fibrin clot. The platelet aggregation in titanium test tubes was similar to that of the glass test tubes with the superiority of a firmer network structure [52] and a longer resorption rate [53]. On the comparative evaluation of T-PRF and L-PRF as better alternatives for the treatment of intrabony defects, T-PRF was suggested to be a better option. However, soft tissue healing was similar in both [54].

Regarding the stability of the PRF membrane, its ability to release cytokines was explored, and results showed a 28-day release rate within a biological environment [55]. However, there are no precise evaluations on the actual residence time of this fibrin membrane placed with a bone graft during the surgical procedure and the degradation effect of enzymes that could impact its efficacy as an autologous barrier. This desired clinical application indicates the need for further development of protocols aiming to modulate stability without loss of controlled release of cytokines from the blood at the site of surgery. Furthermore, another form of purified protein that is easy to isolate from blood plasma precipitation is serum albumin. It has been used in tissue engineering as a result of its compatible structure for cell proliferation and fewer dimensional and degradative changes over time [56]. A preliminary study on the addition of albumin to platelet concentrate indicated that the production of the Alb CGF membrane may represent an important step toward the development of autologous moldable and stable biomaterials for use as soft tissue barriers and potential for different applications in periodontal regeneration [57]. Recent work by Fujioka-Kobayashi et al. (2020) reported that Alb-PRF has positive regenerative properties when used as a low-substitution platelet concentrate with extended resorption properties. The authors also commented that the method of application could either be either an injectable form or with a scaffold by preforming in customized trays to create the desired shape that enhances the long-lasting growth factor release curve capable of stimulating tissue regeneration over extended periods of time [58]. Another concern regarding PRF is its availability in dense gel or solid form, which questions its manipulative potential. Therefore, injectable PRF (i-PRF) was developed [59]. This variant possesses a three-dimensional fibrin clot network embedding platelets, leukocytes, type I collagen, osteocalcin and growth factors acting as a dynamic gel with additional release of growth factors for up to 10 days [60]. A study on rats using an experimental periodontitis i-PRF model resulted in outcomes comparable to scaling and root planing, i.e., reduction in bone loss, modulation of the inflammatory process and cytokine release during the disease process [61]. Table 1 provides a detailed summary of the application of bone grafts with/without PRF.

## 4. Recent Advances in Biomaterials for Periodontal Regeneration

Technological advances of the past decade have fueled innovations in biomaterial design and architecture for periodontal tissue engineering. Application of nanotechnology, with a particle size of less than 100 nm, closely mimics the native structure and architecture of tissues with improved biological properties and bioactivity, leading to a significant increase in regenerative capability [78,79,80,81].

**Figure 2:**
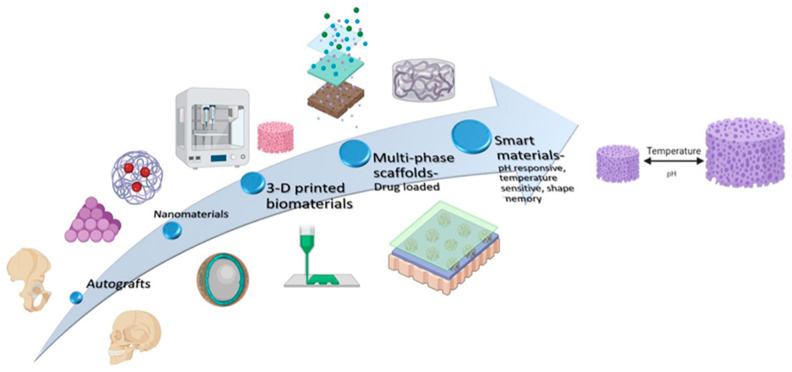
Evolution of biomaterial design and construction used for periodontal regeneration.

The advent of bioprinting has led to biomaterials of a customized three-dimensional structure specific to the defect requirements, which can enhance the form and function of biomaterial. Newer fabrication techniques have enabled biomaterials of different structures and characteristics to be arranged as composite entities that can mimic different kinds of tissues for regeneration of histologically complex anatomies such as periodontal attachment apparatus. Moreover, the incorporation of such materials into a single construct has given rise to composite biomaterials that can release drugs and growth factors, where the release can be controlled in space and time. This section summarizes the novel applications and advancements in ceramics, polymers, metals and composite biomaterial constructions used for periodontal regeneration. (Figure 2).

### 4.1. Innovative Biomaterials Used as Bone Substitutes

#### 4.1.1. Egg-Shell-Derived Nanohydroxyapatite

Egg shells contain 90% calcium and can be used to extract hydroxyapatite for bone regeneration [82]. This biomaterial can be easily procured and is inexpensive with limited drawbacks of patient morbidity and inadequate volume of autografts, possibility of disease transmission in allografts, immune reaction from xenografts, etc. [83]. Recent studies evaluating the efficacy of egg-shell-derived nanohydroxyapatite for bone regeneration in animal models successfully demonstrated its potential to serve as a biomaterial for bone regeneration [84,85]. Furthermore, clinical studies that applied egg-shell-derived nanohydroxyapatite in treating apicectomies, mandibular third-molar defects and socket preservation showed promising results in remodeling and regeneration of bone, which was comparable to synthetic hydroxyapatite [86,87,88].

#### 4.1.2. Metal-Ion-Doped Nanohydroxyapatite

Recently, in an attempt to improve the mechanical and biological properties of hydroxyapatite, few studies investigated the effect of replacing the calcium, phosphate or hydroxyl ions of apatite crystal with different trace metal ions. Comprehensive findings of these experiments revealed:

- Strontium-doped hydroxyapatite showed increased osteoblast proliferation and differentiation [89,90].
- Zinc-doped hydroxyapatite induced differentiation of mesenchymal stem cells to osteoblasts, migration and proliferation of endothelial cells and antimicrobial activity against *Staphylococcus aureus* [91,92].
- Silver-doped hydroxyapatite demonstrated enhanced antibacterial activity and osteoblast adhesion when silver was used in low concentrations; higher concentrations of silver had a negative effect on osteoblast cell proliferation [93,94,95].
- Silicon-doped hydroxyapatite showed significantly greater bone in-growth and bone implant coverage than undoped hydroxyapatite [96].

All these observations indicated that ion-doped hydroxyapatite crystals have enhanced biological properties and can be a promising alternative to pure hydroxyapatite-based bone substitutes.

### 4.2. Magnesium Bone Substitutes

Magnesium is a highly bioresorbable metal with excellent biocompatibility. The magnesium–strontium alloy exhibited favorable characteristics when used as a bone substitute, considering its mechanical properties that resembled natural cortical bone. This alloy had good cytocompatibility and antibacterial and physical properties compared to existing bone graft materials indicating its potential for use as a bone defect filler in stress-bearing areas [97].

### 4.3. Carbon Nanomaterials

Carbon nanotubes are elongated cylindrical nanostructures with a hexagonal honeycomb lattice. Their structure and scale simulate the collagen fibers present in the connective tissue [98]. These nanotubes are porous with a greater surface area when compared to their volume, which facilitates adhesion of cells, adsorption of proteins and delivery of growth factors and drugs [99,100]. Moreover, their superior physical properties enable them to serve as scaffolds for bone regeneration [101]. Multiwalled carbon nanotubes enhanced cementoblast differentiation and mineralization in vitro [102]. Nevertheless, carbon nanotubes, when used as a grafting material in class 2 furcation defects of dogs, showed significantly lesser bone formation when compared to a control group where no grafting material was placed [103]. Further studies are needed to explore the possible applications of carbon nanotubes in periodontal regeneration.

### 4.4. Titanium Bone Substitutes

Titanium is a highly biocompatible metal with extensive applications in biomedical devices. Porous titanium granules offer several advantages as a bone defect filling material, such as superior physical properties for better osseoconduction, high porosity facilitating absorption of blood [104] and high surface area, leading to activation of blood coagulation on contact and subsequent platelet aggregation and adsorption of plasma proteins onto the surface [105]. In an animal study, porous titanium granules led to greater bone fill when compared to deproteinized bovine bone mineral in grade 2 furcation defects [106]. However, there was no significant difference in bone gain in furcation defects in human study [107].

## 5. Innovations in Biomaterials Used for Guided Tissue Regeneration

Guided tissue regeneration (GTR) membranes are used for selectively excluding the epithelium and the connective tissue from the periodontal defects, which allows the periodontal ligament cells to proliferate and repopulate the root surface.

### 5.1. Electrospinning

Electrospinning is a technique to produce nanoscale diameter fibers from a polymeric solution. The polymeric solution is loaded into a syringe pump and will be ejected from a blunt tip needle (spinneret), producing a droplet at the tip of the needle. On application of a high-voltage current, the shape of the droplet changes to a cone shape (Taylor cone); an electrified jet of polymer is ejected out, which solidifies into fine threads that are then collected on the grounded collecting plate [108,109,110]. This technique can be used to fabricate both unwoven fibers and spatially aligned fibers. A separate core with an outer shell structure can be fabricated using coaxial spinning; this multilayered structure can be used for manufacturing functionally graded and drug-loaded membranes. The nanofibers can also be customized by adding drugs, growth factors or ceramics to the initial polymeric solution to create a composite matrix [111,112].

Electrospun poly (d, l-lactic acid)/poly (d, l-lactic-co-glycolic acid) membranes showed superior physical properties, optimum degradation rate and excellent cell occlusion properties, both in vitro and in vivo [113]. Recently, a composite membrane of electrospun polycaprolactone reinforced with 2% bioactive glass showed superior physical and mechanical properties with enhanced cell adhesion [114]. Electrospun polylactic acid/cellulose acetate (PLA/CA) or poly(caprolactone) membrane incorporated with silver nanoparticles showed enhanced antibacterial activity; the addition of nanohydroxyapatite improved the cell viability [115]. Another recent study showed that electrospun poly (L-lactic acid) (PLA)/gelatin membranes incorporated with magnesium oxide nanoparticles showed enhanced mechanical and antibacterial properties, induced osteogenic differentiation of bone marrow mesenchymal stem cells and showed superior outcomes in periodontal regeneration in a rat model [116]. Electrospun zein/gelatin/nanohydroxyapatite membranes showed increased adhesion, proliferation and osteogenic differentiation of human periodontal ligament stem cells [117].

#### 5.1.1. Antibacterial GTR Membranes

Recently, GTR membranes with added antimicrobial functions have been fabricated and evaluated for periodontal regeneration. Expanded polytetrafluoroethylene (e-PTFE), collagen and glycolide fiber membranes loaded with tetracycline or amoxicillin showed less adhesion and penetration of *S. mutans* and *A. actinomycetemcomitans* [118,119]. Electrospun zein/ethyl cellulose membrane loaded with indomethacin showed sustained drug release [120]. Doxycycline-loaded poly-e-caprolactone membranes showed sustained release of the drug and significant inhibitory effect on *A. actinomycetemcomitans* and *P. gingivalis* [121]. Amoxicillin-loaded, electrospun polylactic acid nanofiber membranes showed antibacterial activity against *Streptococcus sanguinis* and *Porphyromonas gingivalis*, control of inflammation and promoted periodontal ligament cell migration in rat periodontal defects [122].

#### 5.1.2. Functionally Graded, Multilayered GTR Membranes

Functionally graded membranes have multiple layers; each layer is fabricated to be closely associated with a specific type of tissue in order to control and dictate its behavior, facilitating guided tissue regeneration. Recently, a novel trilayered chitosan membrane with varying concentrations of bioactive glass among the layers was prepared and evaluated. The lower layer, which was designed to interface with the bone, was porous, hydrophilic and had the highest concentration of bioactive glass; this layer showed osteoblast and fibroblast cell adhesion and proliferation. The upper layer, designed to interface with the connective tissue and the epithelium, was nonporous, hydrophobic and had no bioactive glass and showed no cell adhesion and proliferation [123]. Similarly, a dual-layered membrane containing fish collagen and poly-vinyl alcohol (PVA) showed bone marrow mesenchymal stem cell adhesion and differentiation into osteoblasts on the collagen layer, whereas the PVA layer did not facilitate stem cell adhesion [124]. Another trilayered membrane containing chitosan, polycaprolactone and gelatin showed superior mechanical stability, enhanced blood clotting, biocompatibility and cell exclusion properties, indicating its potential in guided tissue regeneration [125].

## 6. Innovations in Biomaterial Construction and Design for Tissue Engineering

The periodontal attachment apparatus is an anatomically and histologically complex zone containing different types of tissues integrated together for the purpose of supporting the teeth in the jaws: the periodontal ligament (a fibrous tissue) connects the bone to the cementum of the tooth root; the entire complex is covered by epithelium and connective tissue of the gingiva. To be able to regenerate the periodontal complex in its entirety, there is a need to customize biomaterials that contain different parts or zones that would mimic and regenerate each type of tissue. The construction of such complex scaffolds has become a reality due to the application of novel biomaterials, innovative engineering solutions and fabrication techniques that can combine different biomaterials.

### 6.1. Trilayered Nanocomposite Hydrogel Scaffold

**Figure 3:**
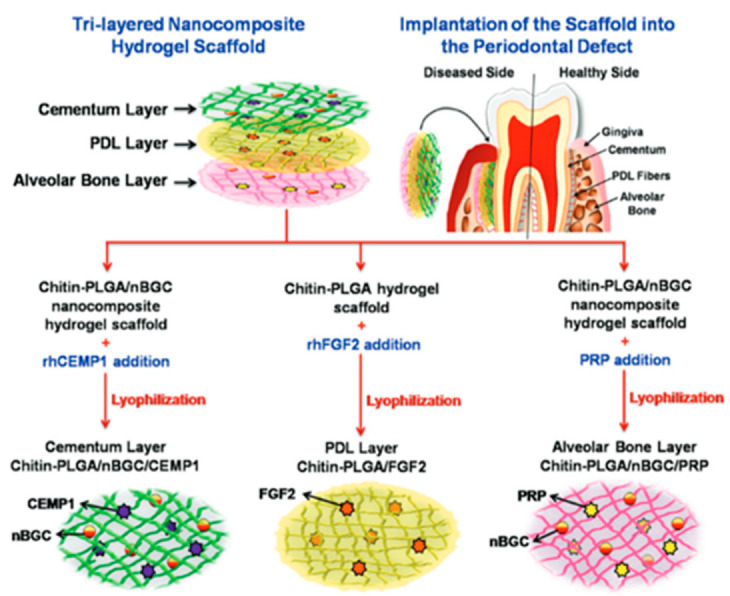
Trilayered nanocomposite hydrogel scaffold for periodontal regeneration. “Reprinted (adapted) with permission from Ref. [126]. Copyright 2017 WILEY-VCH Verlag GmbH & Co. KGaA, Weinheim.

An in vitro study conducted in 2017 evaluated a nanocomposite hydrogel scaffold with three distinct layers; each layer was designed to regenerate a specific type of tissue in the periodontium. The cementum layer was made of chitin-poly (lactic-co-glycolic acid) (PLGA) hydrogel scaffold with nano-bioactive glass ceramic and cementum protein-1; this layer faces the root surface. The alveolar bone layer that faces the bone surface was made of a chitin-PLGA hydrogel scaffold with nano-bioactive glass ceramic and platelet-rich plasma (PRP). The middle layer attempting to regenerate the periodontal ligament fibers was made of the same chitin-PLGA hydrogel scaffold but contained fibroblast growth factor (FGF). The three layers were assembled together to form a biocompatible composite scaffold, which induced differentiation of human dental follicle stem cells into osteoblasts, cementoblasts and fibroblasts (Figure 3). The implantation of the scaffold in a rabbit periodontal defect caused complete regeneration of the periodontium; histological and immunohistochemical analysis confirmed the formation of new periodontal ligament, cementum and bone [126].

#### 6.1.1. Sandwich Tissue-Engineered Complex

A sandwich complex with three layers was fabricated into which gingival fibroblasts were seeded on both sides of a collagen membrane and cultured for 3 days to create a tissue-engineered membrane. This membrane formed the central layer of the sandwich. The outer layers were formed by a layer of the intestinal mucosa (porcine jejunum) seeded on one side by gingival fibroblasts; they were cultured and placed in a mineralization induction medium to form a mineralized membrane. These mineralized membranes formed the outer layers on both sides of the tissue-engineered membrane to form the sandwich construct. The mineralized membranes attempted to regenerate the hard tissues (bone and cementum), and the tissue-engineered membrane attempted to regenerate the periodontal ligament. The implantation of these sandwich constructs led to complete regeneration of the periodontal defects in beagle dogs’ experiment; histological analysis confirmed the formation of new bone, cementum and periodontal ligament within 10 days [127].

#### 6.1.2. 3D-Printed Multiphase Scaffold

Three-dimensional (3D) printing technology was used to assemble a poly-caprolactone-hydroxyapatite scaffold in three distinct phases: Phases A, B and C were designed with 100 μ, 600 μ and 300 μ microchannels incorporated with PLGA microspheres encapsulating amelogenin (to regenerate cementum/dentin), connective tissue growth factor (CTGF) (for periodontal regeneration) and bone morphogenetic protein-2 (BMP-2), (for bone regeneration). This multiphase scaffold was then seeded with dental pulp stem cells (DPSCs) and cultured in vitro before implanting them subcutaneously in immunodeficient mice. After 4 weeks of implantation, histological analysis showed mineralized tissue formation on Phases A and C; Phase B contained collagen fibers inserted into mineralized tissues and fibroblast-like cells along with blood vessels. The PLGA microspheres were used to deliver biological growth factors for stem cell differentiation; the release of the growth factors was controlled in time over 6 weeks [128].

### 6.2. Temporal Control of Drug Release

A chitosan scaffold containing alginate microparticles loaded with insulin-like growth factor-1 (IGF-1) and PLGA nanoparticles loaded with bone morphogenetic protein-6 (BMP-6) was fabricated to study the temporal sequence of drug release. The results exhibited initial rapid release of the alginate microparticles, and since PLGA nanoparticles have a longer degradation rate, they release BMP-6 in a sustained manner. Hence, this system was used to control the temporal sequence of drug release in a phased manner. Results showed enhanced proliferation and differentiation of cementoblasts and increased extracellular matrix (ECM) synthesis in vitro [129]. A 2015 study developed a nanosphere in a microsphere strategy to control the release of growth factors. A heparin-gelatin nanosphere is synthesized and loaded with BMP-2; the heparin binds to BMP-2 and protects it from degradation and sustains its release. This nanosphere is then encapsulated into a microsphere. Since the microsphere is made from nanofibers, it mimics the natural structure of ECM and increases cell adhesion and proliferation. This system ensured sustained and controlled release of BMP-2 from the nanosphere and caused significant new bone formation in a rat calvarial defect model, which was confirmed by histological analysis [130].

### 6.3. An Injectable, Immunomodulatory Biomaterial

Hu et al. (2018) developed a novel immunomodulatory biomaterial containing interleukin-4 (IL-4) for switching proinflammatory M1 macrophages to proresolving M2 macrophages in a diabetic rat model. Heparin-modified gelatin nanofibers were self-assembled into a microsphere into which IL-4 was loaded. Heparin binds to IL-4, protecting it from degradation and sustaining its release. When injected into a rat fenestration defect model, it caused switching of macrophages to proresolving M2 phenotype and resolved the inflammation; there was enhanced osteogenic differentiation and new bone formation. This study presented a strategy of host-modulation of periodontal regeneration using biomaterial-controlled release of anti-inflammatory cytokines [131].

### 6.4. Smart Biomaterial with Shape Memory

**Figure 4:**
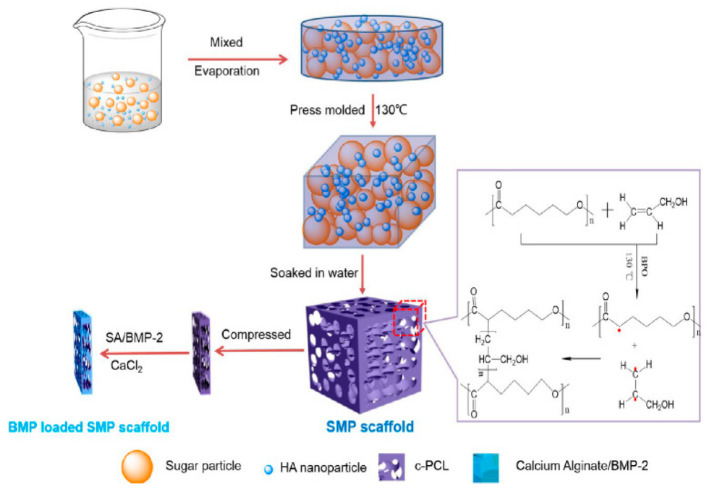
Smart biomaterial with shape memory for periodontal regeneration. “Reprinted (adapted) with permission from Ref. [132]. Copyright 2014 American Chemical Society.

A temperature-sensitive, shape-memory biomaterial was synthesized using cross-linked poly-caprolactone with hydroxyapatite nanoparticles loaded with BMP-2 (Figure 4). This large porous scaffold was deformed into a compressed shape and introduced into a bone defect through a minimally invasive surgical approach; when exposed to body temperature (37 °C), the scaffold regained its original shape to accurately fit into the defect. This shape memory was confirmed using micro-CBCT in a rat mandibular defect model. The scaffold also was shown to be biocompatible and induced significant new bone formation. This study presents an innovative strategy that has great potential in tackling periodontal defects of complex shapes and sizes [132].

## 7. Current Frontiers and Future Horizon

Our current understanding of the wound healing and resolution of inflammation in periodontal tissues is inadequate to successfully manage and treat periodontitis. A complete and thorough understanding of the etiopathogenesis of the disease along with the healing mechanisms are key for engineering biomaterials for periodontal regeneration.

One of the most important problems in managing periodontitis is regenerating the lost tissues in horizontal/supra-alveolar bone defects. In spite of advances in biomaterial engineering, there is a lack of solutions in sight for regenerating tissues in horizontal bone defects that are non-contained and lack spatial control of biomaterials. Newer biomaterial engineering approaches need to emerge to successfully exclude the epithelium and connective tissue from the horizontal bone defect and to spatially orient the biomaterials in the defect area.

An important tenet for the success of periodontal therapy is regenerating all three types of tissues (cementum, periodontal ligament and bone) in the periodontal defect. Even though composite multilayered biomaterial constructions have successfully demonstrated the formation of all three types of tissues in vitro and in experimental periodontal defect models in animals, their efficacy needs to be evaluated in humans. Currently, platelet concentrates show promise in delivering autologous growth factors directly to the periodontal defect. However, the scientific literature reveals numerous protocols for different types of platelet concentrates. Periodontal regeneration is a rapidly evolving, dynamic field with an enormous potential for research.

With advances in biomaterial science and growing interest in tissue engineering strategies, the future displays possibilities for complete rehabilitation and regeneration of the periodontium. Taken together, the combination of bone graft substitutes and stimulatory effects of bioactive materials (growth factors, bone morphogenetic proteins) provide a better outcome in terms of tissue and bone regeneration. The success of tissue-engineered bone regeneration is influenced by various factors that include the use of appropriate scaffolds/constructs for harvesting cells at the defect site, suitable cell type, adequate vascularization, signaling molecules for osteogenic differentiation, etc. Hence, a multidisciplinary approach will be required to develop newer graft materials that possess enhanced properties to obtain more desirable results.

Advancements in scaffold fabrication technologies such as 3D printing, multiphase scaffold, smart biomaterials, etc., have been developed with attempts to regenerate different tissues of the periodontium. Several in vitro studies using these constructs incorporated with nanohydroxyapatite, immunomodulatory biomaterial, gingival fibroblasts, growth factors, etc., have yielded beneficial results. However, clinical trials are required to assess the safety and efficacy of these biomimetic materials for periodontal tissue engineering and bone regeneration purposes in humans.

## Figures and Tables

**Table 1:** Detailed summary of the application of bone grafts with/without PRF.

Author	Biomaterial	Model Used	Observation Period	Outcomes
Ozawa et al. (2018) [62]	Collagen sponge (ACS) hydroxyapatite/collagen composite (HAP/Col)	Rats (male)	12 weeks	Results suggested that application of HAP/Col increased outgrowth of new bone much more prominently than collagen group
Leventis et al. (2018) [63]	β-TCP + poly(lactic-co-glycolic) acid (PGLA) + Biolinker^®^ (N-methyl-2-pyrrolidone solution)	Landrace (female) pigs	12 weeks	Experimental sites showed less mean horizontal dimensional reduction of alveolar bridge but not statistically significant, with more new bone in experimental group
Kizildağ et al. (2018) [64]	Leukocyte-platelet-rich fibrin (L-PRF) + (OFD)/OFD alone	16 humans with 32 sites	baseline and 6 months	(L-PRF) + (OFD) group showed significant PD reduction and CAL gain than OFD alone group
Okada et al. (2019) [65]	Group 1: β-TCP Group 2: β-TCP + PGLA	Beagle (male) dogs	12 weeks	β-TCP + PGLA seems to be more effective than conventional β-TCP for ridge preservation
Sapata et al. (2019) [66]	Deproteinized bovine bone mineral (DBBM) DBBM with 10% collagen–collagen matrix (CM)	65 patients	4 months	DBBM demonstrated a noninferiority status compared to DBBM-CM group
Bodhare et al. (2019) [67]	Control: OFD + BioGide Test: OFD + BioGide + PRF	40 human sites	6 months	BioGide when used in combination with PRF is found to be more effective in gain in CAL, reduction in PD and achieving greater bone fill as compared to treatment with BG alone
Atchuta A et al. (2020) [68]	Group I: open-flap debridement; Group II: DFDBA alone; Group III: DFDBA + PRF	39 human sites	Baseline, 3 months and 6 months	DFDBA + PRF group yielded better reduction of PPD and Relative attachment level (RAL) at 6 months interval
Kai-Ning Liu et al. (2020) [69]	Control group, GTR and Bio-Oss^®^ Test group, GTR, Bio-Oss^®^ and PRF	14 patients	6, 12 and 24 months	GTR and Bio-Oss^®^ with PRF is more effective in treatment of periodontal intrabony defects than GTR and Bio-Oss^®^ without PRF (CAL, PD) at all time intervals
Thakkar B et al. (2020) [70]	Group I: PRF and GTR Group II: PRF + bovine bone graft + GTR	32 human sites	Baseline, 3 months and 6 months	Group II showed statistically significant changes in reduction in pocket depth and defect depth resolution
Bahammam MA et al. (2020) [71]	Group I: PRF + OFD Group II: nano-HA bone graft + OFD. Group III: OFD + PRF + nano-HA bone graft. Group IV: OFD alone	60 human patients	Baseline and 6 months	Most significant increase in bone density and fill was observed for IBD depth in group III
Apine AA et al. (2020) [72]	Group I: NovaBone^®^ putty. Group II: autologous platelet-rich fibrin	30 intrabony defects were treated in 11 patients	Baseline, 3, 6 and 9 months	Improvement of clinical and radiographic parameters at sites treated with NovaBone^®^ putty was better compared to that of sites treated with PRF, but differences were statistically not significant
Paolantonio M et al. (2020) [73]	Test group: L-PRF associated with autogenous bone graft (ABG) Control group: EMD + ABG	44 patients	Baseline and 12 months	L-PRF + ABG produces noninferior results for CAL gain, PPD reduction, GR increase and DBL gain in comparison with EMD + ABG when treating noncontained IBDs.
Jae-Hong Lee et al. (2021) [74]	Test group: demineralized porcine bone matrix (DPBM) with EMD Control group: DPBM alone	34 patients	Baseline, 2 years and 4 years	clinical, radiographic and patient-reported outcomes were significantly improved when DPBM no additional clinical and radiographic benefits were observed with adjunctive use of EMD
Bhatnagar S et al. (2021) [75]	Control sites: OFD alone Test sites: Calcium Phosphate ceramic GUIDOR^®^ easy-graft Crystal, Sunstar Group, Etoy, Switzerland) and OFD.	15 patients; 30 intrabony periodontal defects	Baseline, 3 and 6 months	Significant increase in Defect Fill and Percentage of Defect fill in both groups with better bone fill in test sites
Pavani MP et al. (2021) [76]	Group A: open-flap debridement (OFD) Group B: OFD with β TCP with PRF Group C: β TCP	30 human sites	6 months	Bone fill achieved in β TCP with PRF was more compared to β TCP alone and OFD at 6 months follow-up
Razi MA et al. (2021) [77]	Group I: PRF with demineralized bone matrix Group II: PRF alone Group III: open-flap debridement (OFD)	30 patients	9 months	PRF group had significant reduction in PD, RAL and Gingival recession (GR)

## Data Availability

Not applicable.
